# Fascination of Serpents

**Published:** 1846-06

**Authors:** J. Comstock


10. *Fascination of Serpents.*—" The serpent's power to charm is regarded with scepticism by a great many, but there are very many authentic instances on record. In Williams' History of Vermont, a high authority, you will find some very interesting facts and comments on this subject; but a case has come within my own knowledge which is worthy of publication and may throw some light upon it. It has generally been believed to be the fascination of the serpent's eye. This may have some effect, for probably there is no living eye which has such piercing brilliancy and fascinating beauty; but I have seen little birds under the spell, fluttering about the snake and drawing gradually, like the infatuated votary of vice, to its deadly tempter. It cannot be this altogether. The snake at such times keeps its head vibrating, its forked tongue darting, and its tail trembling, while the whole body moves like that of a creeping caterpillar. The case alluded to above was related to me by Nehemiah Gallup, a revolutionary veteran who died about a year since, in Groton, Ct. He said that, in the revolutionary war, when attached to Fort Griswold, in that town, opposite to New London, he, in company with a number of other soldiers, went out on a hunting excursion, and finding a rattle snake, some of which are occasionally killed in that town, they fixed their bayonets, and forming a circle amused themselves by teasing him, till they all began to grow giddy and sick, when they killed him. They went on their way, thinking no more about it, but gradually grew worse, and on reaching their quarters were so seriously indisposed as to require medical advice; being troubled with excessive nausea at the stomach and vomiting. The physician made particular inquiry in reference to their food, &c., for some time previous, when one of them accidentally told of their adventure with the snake. He at once replied that he was no longer at a loss to account for their sickness, and inquired if they perceived any peculiar odor at the time. They each recollected that they did. He replied, 'I have seen on the lines in the State of New York many instances of this kind. That snake was charming you with a stupefying effusion which they emit at pleasure, and had you not despatched him as you did, probably he would have despatched some of you.' He gave emetics and they recovered. 'Many years afterwards,' said Mr. Gallup, 'I went into a room where two rattle snakes were exhibited, and immediately on entering the room, perceived the same odor, though not so strong, and was so sick that I had to leave the room.' I have never seen this idea advanced by any one else. It seems more reasonable than the other and is worthy of consideration."—J. Comstock, in *Boston Medical and Surgical Journal*, May 6.

